# Novel insights into the mechanism of cell-based therapy after chronic myocardial infarction

**DOI:** 10.15190/d.2014.1

**Published:** 2014-01-30

**Authors:** Alexander Schuh, Britta Butzbach, Adelina Curaj, Sakine Simsekyilmaz, Octavian Bucur, Isabela Kanzler, Bernd Deneke, Simone Konschalla, Andreas Kroh, Tolga Taha Sönmez, Nikolaus Marx, Elisa A. Liehn

**Affiliations:** Department of Cardiology and Pulmonology, Medical Faculty, RWTH Aachen University, Germany; Institute for Molecular Cardiovascular Research (IMCAR), RWTH Aachen University, Germany; Department of Experimental Molecular Imaging, RWTH Aachen University, Germany; "Victor Babes" National Institute of Pathology, Bucharest, Romania; Department of Pathology, Harvard Medical School and Beth Israel Deaconess Medical Center, Boston, MA, USA; Department of Molecular Cell Biology, Institute of Biochemistry of the Romanian Academy, Bucharest, Romania; Institute of Biochemistry and Molecular Cell Biology, RWTH Aachen, Germany; Department of Cardiothoracic and Vascular Surgery, Johann Wolfgang Goethe-University, Frankfurt/Main, Germany; Interdisciplinary Centre for Clinical Research (IZKF) Aachen, RWTH Aachen University, Aachen, Germany; Department of Surgery, University Hospital Aachen, Germany,; Department of Oral and Maxillofacial Surgery, University Hospital Aachen, Germany

**Keywords:** apoptotic bodies, cell therapy, myocardial infarction, transplantation, mechanisms, microRNAs

## Abstract

Cell transplantation therapy is considered a novel and promising strategy in regenerative medicine. Recent studies point out that paracrine effects and inflammation induced by transplanted cells are key factors for the improvement of myocardial function. The present study aims at differentiating paracrine effects from inflammatory reactions after cell transplantation. Therefore, in vitro induced apoptotic bodies were transplanted after myocardial infarction in a rat model. Eight weeks after transplantation, the functional results showed no improvement in left ventricular function. Histological analysis revealed no significant differences in the amount of infiltrated cells and collagen content did not differ among the four groups, which sustains the functional data. Surprisingly, angiogenesis increased in groups with apoptotic bodies derived from HUVEC and endothelial progenitor cells, but not from fibroblasts. A complex genetic analysis of apoptotic bodies indicated that miRNAs could be responsible for these changes. Our study demonstrates that inflammatory reaction is critical for scar remodelling and improvement of the heart function after late cell therapy, while neoangiogenesis alone is not sufficient to improve heart function.

## Introduction

Due to the modern predominance of sedentary, yet stressful lifestyles, atherosclerosis remains the most common disease afflicting humans worldwide^1^. Its most feared complication, acute myocardial infarction (MI), continues to be the leading cause of mortality despite considerable efforts and numerous advances in the diagnosis and management of the disease.

The ischemic cardiomyopathy following myocardial infarction is induced by the loss of functional myocardial tissue. After the necrotic tissue in the affected area is replaced with fibrotic tissue, the remaining myocardial tissue is overcharged and consumes all its reserves, triggering heart failure^2^. An important goal of several previous studies has been to elucidate the pathomechanisms of myocardial tissue regeneration in postischemic myocardium. Stem-cell based therapy is a novel strategy in regenerative medicine whose ultimate goal in cardiac repair is to regenerate healthy, functionally integrated myocardial tissue. Many experimental animal studies have already shown significant improvement in heart function after transplantation of different stem or progenitor cells^3,4^. The method employed has been to integrate transplanted cells into the infarction site, thereby increasing the contractile tissue^5-7^ or enhancing the neovascularisation^8-10^.

However, implementation of cell therapy techniques in clinical have not delivered the expected results^11,12^. The most common factor limiting the efficacy of cell-based therapy has turned out to be poor survival rates of transplanted cells in an ischemic environment. Despite the fact that some cells were found to integrate into the host tissue, we have already observed in our research that most of the transplanted cells undergo apoptosis^8^. Therefore, this mechanism cannot entirely explain the improvement of the heart function in animal studies and may be one reason that the animal-human translational methods have proved to be inadequate for use by clinics.

In recent years, the inflammatory reaction induced by transplanted cells has been demonstrated as a viable mechanism for improving heart function after cell therapy^13^. Fibroblasts as well as inactive glass microspheres were able to increase the contractility of the ventricular wall significantly by initiating an inflammatory reaction^13^. Inflammatory cells, neutrophils and monocytes secrete proteases, cytokines and chemokines, which change the surrounding environment^1^ and thus improve the heart’s functional parameters^13^.

On the other hand, depending on the type of cell transplanted, variations in the infarction scar could be observed, including angiogenesis and changes in collagen content. We hypothesized that the transplanted cells undergoing apoptosis might also be able to influence the surrounding environment in that they release apoptotic bodies, which are small sealed membrane vesicles containing active factors capable of exerting paracrine effects^14-16^. Together with the inflammatory reaction, these could be the key mechanisms for achieving sustained improvement of myocardial function after cell therapy. However, until now, it has not been clear which ones of the observed effects in the recipient heart are due to inflammation and which ones are due to the inflammation-independent paracrine effects exerted by the transplanted cells on their surroundings (such as on induction of angiogenesis).

The most common limiting factor has turned out to be poor survival rates of transplanted cells in an ischemic environment. Despite the fact that some cells were found to integrate into the host tissue, we have already observed in our research that most of the transplanted cells undergo apoptosis. Therefore, this mechanism cannot entirely explain the improvement of the heart function in animal studies. This may be one of the reasons why the animal-human translational methods have been proven to be inadequate for use by clinicians.

In this study we therefore induced apoptosis in several types of cells *in vitro* and transplanted only apoptotic bodies. Since apoptotic bodies are phagocytosed by surrounding cells and do not physiologically induce inflammation, we would be able, after transplantation, to analyze manly the effects induced by the paracrine stimulation. This approach will be essential for elucidating cell-mediated regenerative mechanisms, improving cell-based therapy, and identifying promising, accessible strategies by which to optimize cardiac repair in current clinical practice.

## Materials and Methods

### Rodent model of myocardial infarction

Adult female Sprague Dawley rats (200-250 g) were intubated under general anesthesia (100mg/kg ketamine and 10 mg/kg xylazine, intraperitoneal). Positive pressure ventilation was maintained with supplemented ambient air using a rodent respirator. A 1 cm left thoracotomy was performed to expose the heart, and the left descending artery (LAD) was tied between the left atrium and the right pulmonary outflow tract using a 7/0 polyprolene snare (Ethicons Products, Norderstedt, Germany). After closing the thorax by binding the ribs with silk suture, the muscle layer and the skin incision were sewn. Animal experiments were approved by local authorities and complied with German animal protection statutes.

### Preparing of cells and apoptotic bodies

Human umbilical veno-endothelial cells were thawed and cultivated as previously described^8^. The cells were grown to confluence before undergoing apoptosis. Human endothelial progenitor cells (EPCs) were isolated from 20 ml of citrate/dextran anticoagulated peripheral blood following previously published protocols^17-19^ and were subjected to apoptosis after culturing for seven days. Confluent human dermal fibroblasts were a gracious donation by the Department of Plastic Surgery and the Pathology Institute at the RWTH-Aachen (Dr. C. Suschek, Dr. S. Neuss-Stein). To induce apoptosis, the cells were incubated with cyclohexamide (5µg/ml) in hypoxia for 24 hours, mimicking the condition of transplanted cells. Afterwards, the apoptotic bodies were collected as previously described in our laboratory^20^. Medium from the apoptotic cells was collected and cleared of debris by centrifugation (800g, 10 min). The apoptotic bodies were then isolated from the supernatant by high speed centrifugation (16,000g, 20 min).

### Apoptotic bodies transplantation

In accordance with our previous studies^8,10,13^, transplantation was performed four weeks after acute myocardial infarction. The rats were anaesthetized and their hearts were exposed by thoracotomy in the manner previously described. The apoptotic bodies were transplanted into the marginal zones of myocardial infarction by syringe injection (one minute injection-time) at three distinct, but adjacent, sites. To isolate the effects of apoptotic bodies and afford comparison with the effects of transplantation of entire cells, the amount of apoptotic bodies transplanted was that derived from the same quantity of cells as we had transplanted in previous studies^8,10,13^. The animals were divided into four groups consisting of 7 or 8 rats per group. The first group was control and received only phosphate-buffered saline (PBS); the second group received apoptotic bodies of HUVEC (HUVEC-AB); the third group received apoptotic bodies of EPCs (EPC-AB); the fourth group received apoptotic bodies of fibroblasts (Fibro-AB). After injection, puncture sites were sutured. Cyclosporin A (50 mg/kg) was applied orally in the groups by daily injection beginning with the day of transplantation.

### Echocardiography

At points in time before myocardial infarction, four weeks after acute myocardial infarction, and two months after trans-plantation, all rats were anaesthetized with iso-flurane, whereupon two-dimensional and m-mode measurements were conducted using a SONOS 5500 HP (Agilent, Palo Alto, CA, USA) with a 12.5-MHz linear phased-array probe. The rats were placed in the supine or lateral position and excessive pressure on the thorax was avoided. Parasternal long-axis and short-axis views were performed, ensuring that the mitral and aortic valves and apex were well visualized and recorded. Measurements of left ventricular (LV) end-diastolic and end-systolic dimensions were conducted in m-mode for more than three beats, and ejection fraction (EF) and fractional shortening (FS) were calculated as previously described^10^.

### Cell identification and analysis of infarct area

Macrophages and neutrophils were stained with alpha-napthyl acetate esterase (Sigma-Aldrich, Germany) or naphthol as-d chloroacetate (Sigma-Aldrich, Germany), respectively. Each heart was analyzed on the basis of either 3 or 4 sections. Cells were counted in either 3 or 4 fields of each section, respectively. Stained cells (macrophages in yellow-brown, neutrophils in red) were counted per mm^2^ of the infarct area. We analyzed neoangiogenesis in infracted areas by counting CD31-positive ring structures on the basis of CD31 antibody (Santa Cruz Biotechnology, Heidelberg, Germany). These values are expressed as an absolute number per mm². Nuclei undergoing apoptosis were stained with MEBSTAIN apoptosis kit II (MBL International, Woburn, MA, USA) and TUNEL-positive (i.e. apoptotic) nuclei were counted. The relevant values were calculated as a percentage of all nuclei (apoptotic index). The collagen content of the infarct-regions was determined using Gomori’s 1-step trichrome staining. The collagen blue-stained areas were measured by computer assisted planimetry (P-Cell Software, Olympus) and expressed as percent of the total infarct area.

### Matrigel experiments

BD Matrigel TM Basement Membrane Matrix (BD Bioscience, Heidelberg, Germany) was used as recommended by the manufacturer. In brief, matrigel was applied to a 96-well plate. HUVECs were cultivated on matrigel and incubated for 24 hours with apoptotic bodies isolated from HUVECs, EPCs, and fibroblasts. VEGF-rich medium was used as positive control and 1% BSA in RPMI was used as negative control. Tube formation was quantified using digital cell culture microscope (AMG Evos, Darmstadt, Germany).

### ELISA

ELISA was performed on apoptotic bodies lysates using DuoSet ELISA Development System kit for vascular endothelial growth factor (VEGF), stromal derived factor (SDF)-1 and keratinocyte-derived chemokine (KC) (R&D Systems, Wiesbaden, Germany), according to the manufacturer’s instructions. The amounts of apoptotic bodies were quantified by measuring their protein content as described in the manufacturer's instructions (Bio-Rad D_C_ Protein Assay Kit; Bio-Rad, Munich, Germany).

### Affymetrix miRNA labelling, array hybridization and data pre-processing

Total RNA was isolated from apoptotic bodies with the ZR RNA MicroPrep kit (Zymo Research, Freiburg, Germany) according to the manufacturer´s instructions. The quantity of total RNA was measured using a NanoDrop ND-1000 Spectrophotometer (NanoDrop Technologies, Wilmington, DE, USA). Optical density values at 260/280 were consistently above 1.7. Total RNA containing low molecular weight RNA was labelled using the Flashtag RNA labelling kit (Genisphere, Hatfield, PA, USA) according to the manufacturer's instructions. In brief, for each sample, 32 ng total RNA was subjected to a tailing reaction (2.5 mM MnCl_2_, ATP, Poly A Polymerase - incubation for 15 minutes at 37°) followed by ligation of the biotinylated signal molecule to the target RNA sample (1× Flash Tag ligation mix biotin, T4 DNA ligase - incubation for 30 minutes at RT) and addition of stop solution. Each sample was hybridized to a GeneChip® miRNA 3.0 Array (Affymetrix, Santa Clara, CA, USA) at 48°C and put on 60 rpm for 16 hours before being washed and stained on Fluidics Station 450 (Fluidics script FS450_0002) and, finally, scanned on a GeneChip® Scanner 3000 7G (Affymetrix, Santa Clara, CA, USA). The image data was analyzed with the miRNA QC Tool software for quality control. For each source of apoptotic bodies, three independent experiments were performed under identical conditions. The expression values were summarized and normalized with robust multi-array average (RMA)^21^ using RMA package in Bioconductor 2.5 under R 2.10^22^. Only miRNAs that were detectable in all three arrays for each group and whose expression levels were found to have changed significantly (P<0.05), at least by twofold, were considered to be differentially expressed. All array data are MIAME compliant. The raw data has been deposited in Gene Expression Omnibus (GEO) - Accession number: GSE48218.

### Statistical analysis

Data represent mean +/-SEM. Data analysis was conducted with Prism 4 software (Graph Pad) using the unpaired Student *t* test or one-way ANOVA followed by Newman-Keuls test. Differences of p**< 0.05 were considered significant. For miRNA analysis we used the empirical Bayes moderated t-statistic^23^, which ranks genes by testing whether all pairwise contrasts between different outcome-classes are zero. An empirical Bayes method is used to shrink the sample-variances for each sample towards a common value and to augment the degrees of freedom of individual variances^23^. For multiclass problems the F-statistic is computed as an overall test of t-statistics for every genetic sample. We use the eBayes-implementation in the R-package limma (v2.12).

## Results

### The effect of transplanting apoptotic bodies on heart parameters after myocardial infarction**

**Figure 1:**
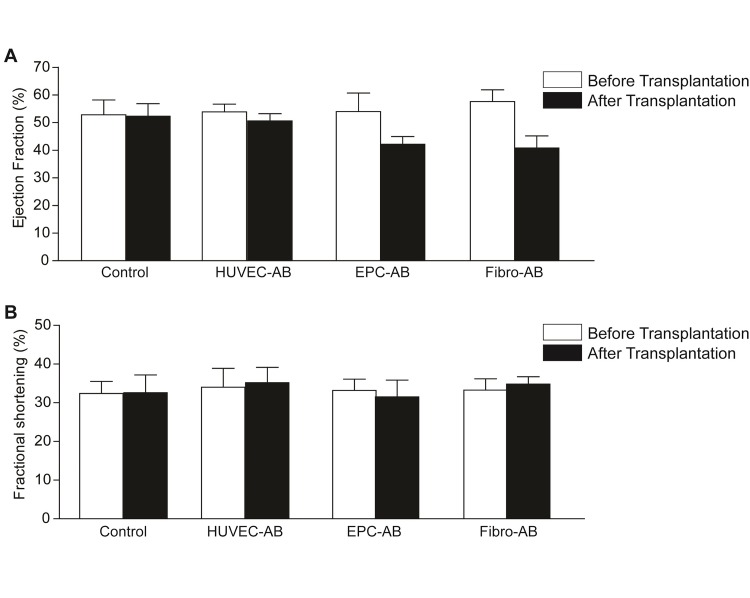
The effect of apoptotic bodies transplantation on heart parameters after myocardial infarction Analysis of the functional parameters of the heart by echocardiography demonstrated no significant improvement of ejection fraction (A) and fractional shortening (B) after apoptotic bodies transplantation in all four groups, before (empty bars) and after transplantation (full bars).

Four weeks after myocardial infarction, apoptotic bodies from HUVEC (HUVEC-AB), EPCs (EPC-AB), and fibroblasts (Fibro-AB) were transplanted into the border zone of the infracted scar. The control group received PBS (Control). Analysis of the infarcted areas revealed no differences in the infarction size among the groups. Moreover, analysis of the hearts’ functional parameters by echocardiography demonstrated no significant improvement in ejection fraction (52.29±4.56% in control group, 50.63±2.62% in HUVEC-AB group, 42.17±2.76% in EPC-AB group, 40.80±4.38% in Fibro-AB group, **Figure 1** [A]) or fractional shortening (32.60±4.57% in control group, 35.20±3.94% in HUVEC-AB group, 31.50±4.33% in EPC-AB group, 34.80±1.93% in Fibro-AB group, **Figure 1** [B]) after transplantation of apoptotic bodies.

### Histological and immunohistochemical analysis of infarcted area after apoptotic bodies transplantation**

**Figure 2:**
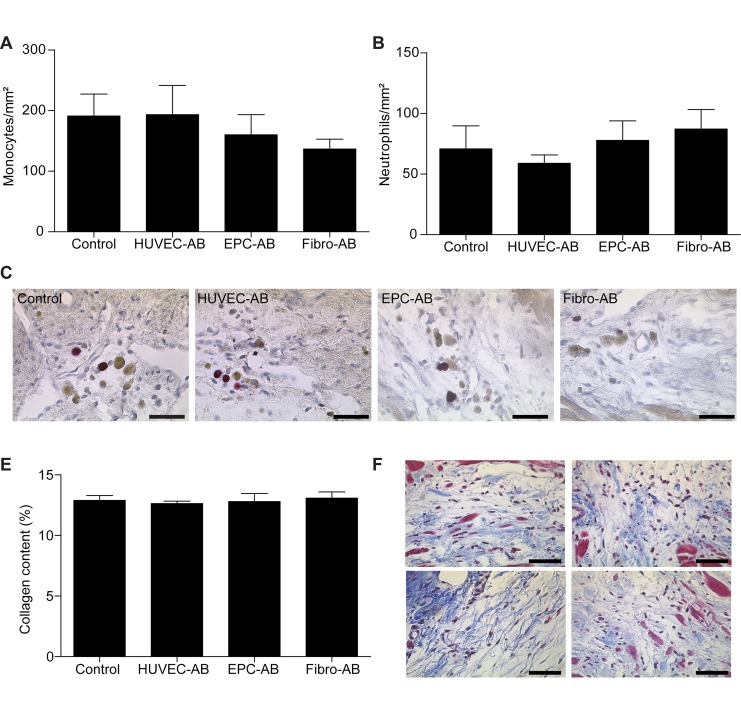
Analysis of inflammatory reaction in the infarcted area The transplantation of apoptotic bodies does not induce significant changes in the monocytes (A, C - brown) and in neutrophils quantification (B, C - red) between the experimental groups. Representative pictures from infarction areas are shown (C, Scale bar 25µm). Collagen content showed no differences between the groups (D). Representative sections of Gomori staining are shown (E, collagen blue, Scale bar 25 µm).

To analyze the inflammatory reaction, the neutrophils and monocytes were quantified in all groups. As expected, the apoptotic bodies were not found to have induced changes in the subpopulation of recruited leukocytes as compared with the control group. The monocyte count (191±36 cells/mm² in control group, 193±48 cells/mm² in HUVEC-AB group, 160±33 cells/mm² in EPC-AB group, 136±16 cells/mm² in Fibro-AB group, **Figure 2** [A]) did not significantly differ from the neutrophil count (70±19 cells/mm² in control group, 59±7 cells/mm² in HUVEC-AB group, 78±16 cells/mm² in EPC-AB group, 87±16 cells/mm² in Fibro-AB group, **Figure 2** [B]) in any group.

Remodelling due to transplantation of the apoptotic bodies was analysed on the basis of measurements of the collagen content of the infarcted scar by Gomoris’1-step trichrome staining. The collagen content did not differ among the groups, which shows that the inflammation-independent effects of paracrine are not sufficient to alter the collagen structure (**Figure 2** [D,E]).

**Figure 3:**
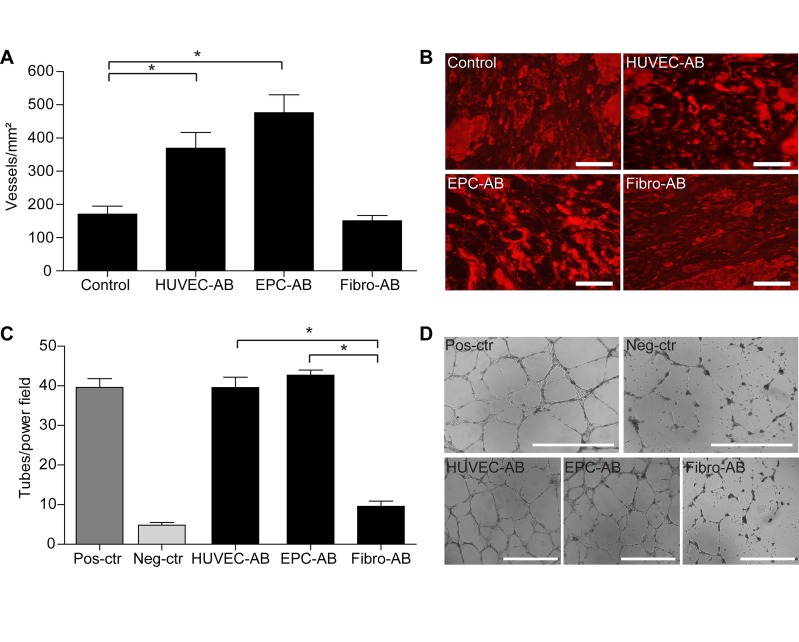
Analysis of angiogenesis in the infarcted areas The quantification of CD31-positive vessels (red) reveals significant differences in the control group compared with EPC-AB and HUVEC-AB group, but not with Fibro-AB group (A, *p<0.05). Representative pictures from infarction areas are shown (B, Scale bar 25µm). *In vitro* matrigel experiments showed that apoptotic bodies from HUVEC and EPCs were able to induce tube formation, but not the apoptotic bodies from fibroblasts (C, *p<0.05). Representative pictures from positive control group (Pos-ctr, incubated with VEGF-rich medium), negative control group (Neg-ctr, incubated with 1%BSA), as well as from EPC-AB, HUVEC-AB and Fibro-AB groups were showed (D, Scale bar 1000µm).

Our finding of significant differences in the density of newly formed vessels, which we assessed by quantifying CD31-positive ring structures in the infarcted area (**Figure 3** [A,B]), was surprising. In the EPC-AB and HUVEC-AB groups, neoangiogenesis was significantly higher than in the control group (476±54 cells/mm² in EPC-AB and 369±47 cells/mm² in HUVEC-AB, vs.170±24 cells/mm², p<0.05).

In the Fibro-AB group, neoangiogenesis did not differ as compared with the control group (150±15 cells/mm² vs. 170±24 cells/mm²). To test the angiogenic potential of apoptotic bodies *in vitro*, we performed matrigel experiments using VEGF-rich medium as positive control and 1%BSA as negative control as described under Methods. As in the *in vivo* results, only the apoptotic bodies from the EPC and HUVEC groups (42.7±2 tubs/field in EPC-AB and 39.6±3 tubs/field in HUVEC-AB, vs. 39.6±2 tubs/field in positive control, **Figure 3** [C,D]) induced tube formation, while those from the fibroblasts did not (9.6±1 tubs/field in Fibro-AB group vs. 4.8±1 tubs/field in negative control, **Figure 3** [C,D]). These results are the first demonstration that, contrary to previous assumption, neoangiogenesis alone is insufficient to improve heart function.

### Molecular analysis of apoptotic bodies**

In order to determine what molecular mechanisms induced the observed significant differences in angiogenesis, we analyzed protein levels of known angiogenic factors such as vascular endothelial growth factor (VEGF), stromal derived factor (SDF)-1, and keratinocyte-derived chemokine (KC) in apoptotic bodies by ELISA. None of these proteins could be detected in the apoptotic bodies derived from the three cell lines analyzed. We do not exclude the existence of other angiogenic factors, however, due to the reduced protein level found in apoptotic bodies, we speculate that they can only play a minor role in inducing angiogenesis.

**Figure 4:**
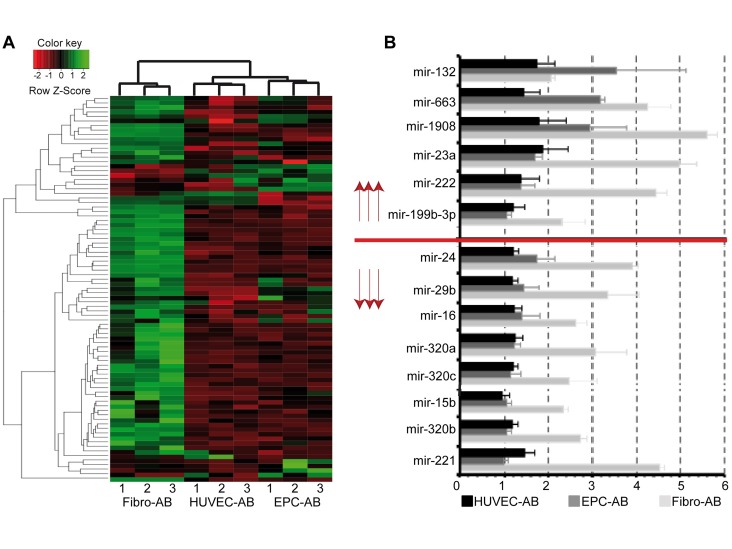
Analysis of angiogenic potential of the apoptotic bodies Further, cluster analysis of miRNAs differently expressed between Fibro-AB, HUVEC-AB, and EPC-AB, respectively was performed (A). Only miRNAs with a fold change of more than 2 between one of the groups with a p-value smaller than 0.05 were selected. Cluster analysis was performed using the eBayes-implementation in the R-package limma (v2.12). Specific colours indicate the row Z-score as indicated in the colour key. We analyzed the expression of angiogenesis related miRNAs (B). Shown is the signal intensity compared with total RNA as log2 of the mean with the S.D. of each three independent array experiments in HUVEC-AB (black bars), EPC-AB (grey bars) and Fibro-AB (hell bars).

Since, DNA-sequences contained in apoptotic bodies are random nuclei fragments and can not have a constant composition, and given what we have learned about microRNAs, small non-coding RNA molecules that are capable of affecting gene expression and are likely involved in most biological processes, we hypothesized that the molecular mechanisms of angiogenesis induction could involve apoptotic bodies exerting paracrine angiogenic effects. Using GeneChip® miRNA, microRNAs (miRNA) were analysed in apoptotic bodies from EPC, HUVEC, and fibroblasts. We found significant differences among the groups in more than 80 miRNA sequences (**Figure 4** [A]). Cluster analysis on the basis of eBayes-implementation in the R-package limma (v2.12) showed profiles in apoptotic bodies derived from EPC-cells (EPC-AB) similar to those derived from HUVEC-cells (HUVEC-AB), while apoptotic bodies derived from Fibroblasts (Fibro-AB) had a completely different miRNA profile (**Figure 4** [A]). To determine whether the observed differences in angiogenesis among the groups might have been conditioned by miRNAs, we analysed which miRNAs have been documented to influence angiogenesis (**Table 1**, **Figure 4** [B]). We found that several miRNAs known to increase or inhibit angiogenesis were expressed in all three cell lines-derived apoptotic bodies. However, whereas the pro-angiogenic miRNAs predominated in EPC-AB and HUVEC-AB, the anti-angiogenic miRNAs predominated in Fibro-AB. Although we can not exclude the involvement of other mechanisms, based on the detection of many miRNAs with yet unknown functions, our results may explain the histological results (**Figure 4** [B]).

## Discussion

Our research aims to elucidate cell-mediated regenerative mechanisms in order to improve the translation of cell therapy from animal to human systems. Previous reports led us to suspect inflammation and paracrine effects of the transplanted cells as the main mechanisms responsible for improving contractility and heart function.

Many studies have attempted to elucidate the mechanisms responsible for the beneficial effects of cell-based therapy following myocardial infarction. However, it seems that attenuation of myocardial dysfunction after cell transplantation results from a combination of different mechanisms. Stimulating neovascularization^10^, attenuating the loss of myocardial function^7^, and modulating inflammatory processes^13^ or paracrine effect^24-28^ were all suspected to be partially responsible for protecting the heart. Cells preconditioned with growth factors or even genetically altered cells have caused additional improvement in myocardial function^29-31^. Nevertheless, different cell types have been thought to improve myocardial function through different mechanisms. Whereas the main mechanism in the case of transplanted endothelial cells or endothelial progenitor cells (EPCs) may be improving neovascularization^8-10^, the main beneficial effect in the case of transplanted myocardial cells (fetal cardiomyocytes or mesenchymal stem cells) seems to be the additional reservoir of contractile cells^7^ they provide.

Unfortunately, the results of clinical implementation of cell therapy have been disappointing^11,12^. Strauer et al^11^, surveying four meta-analyses involving 2,940 patients, considered the 4% increase in ejection fraction significant and sufficient to improve symptoms and reduce mortality in treated patients. This level of improvement, however, remains disappointing. Many possible procedural grounds have been invoked in an effort to explain this failure: the number and method of preparing cells, the manner and timing of their delivery, or the methods of evaluating heart function. But we hypothesized instead that the root cause of the striking discrepancy between experimental and clinical results was not to be sought merely in procedure but ultimately in an incomplete understanding of the molecular mechanisms involved in cell-based therapy. Improved understanding of these would be a prerequisite for fruitful discussion of procedures.

Some authors have speculated that transplanted cells can acquire the functional properties of resident cells. Such consideration has extended to the contractility of myocytes^7^ and to mechanisms of transdifferentiation^32^, fusion^33^, or cytokine-induced support of residual viable myocytes^34^. We have managed to confirm improved contractile function after transplantation of fetal cardiomyocytes^7^. However, a large number of cardiomyocytes is required to reverse left ventricular remodelling and improve cardiac function. Concerning this matter, Reinecke and Murry have demonstrated the risks of tissue overgrowth, which can distort ventricular contour when myoblasts are transplanted as a bolus^35^. Moreover, endothelial progenitor cells (EPCs) do not influence myocyte viability. They are thought to integrate into newly formed vessels and to increase neoangiogenesis. By implication, this should improve heart function^9,10^.

Elsewhere we have demonstratedthat transplanting biologically inactive cells such as fibroblasts as well as glass microspheres (uniform polystyrene, 10 µm diameter) into infarcted myocardial areas improved myocardial function as compared with control hearts^13^. In this regard, we should thus revise the notion that differentiation and integration of cells into host tissue will achieve significant functional improvement. We have found instead that inflammation triggered by transplantation modifies remodelling processes and contributes to improvement of the heart function^13^. Furthermore, standard pharmacological therapies known to modulate inflammatory processes, such as statins and ACE inhibitors, improve heart function in experimental myocardial infarction by cell-therapy similar mechanisms^36^. Moreover, contrary to previous thinking, no differences in the efficiency of early (<2 weeks) versus late cell transplantation (>8 years) have been detected in clinical studies^11^. This supports the view that the inflammatory processes are crucially involved in the improvement of heart function after cell therapy.

However, it seems that inflammation cannot be typified. Previous own studies of ours using late transplantation have involved more monocytes than neutrophils^9,10,13^. Different monocyte subpopu-lations are probably recruited depending on which paracrine factors are released from the cells^37^. This might explain the lack of clinical inflammation parameters (leukocyte count, C-reactive protein, creatine phosphokinase)^11^ as well as the significant effect of injecting monocyte-recruiting factors such as monocyte chemotactic protein (MCP)-1^38^ or stromal derived factor (SDF)-1^39^. Nevertheless, it seems that monocytes themselves (with their secretome) reduce myocardial fibrosis and enhance angiogenesis, thereby attenuating pathological remodelling and preserving functional myocardium after ischemic insult^40^. However, the mechanism is incompletely understood and requires a great deal more investigation.

We have already shown that transplanted cells undergo apoptosis and have suggested that this may be due to the hypoxic and ischemic environment^8^. Before going into apoptosis, the cells can release different soluble factors which can be in part responsible for some observed effects. However, synthesis and release of such proteins by dying cells are hard to be monitored and controlled. Apoptosis affects single cells and is characterized morphologically by nuclear fragmentation, with apoptotic bodies being generated and becoming visible either within dying cells or in the interstitial spaces^41^. The apoptosis process serves to eliminate damaged or unnecessary cells cleanly, without disrupting the surrounding tissue or eliciting an inflammatory response^42^. Apoptotic bodies are poorly understood. They are much smaller than the cells in which they originate, but they can influence cellular pathways and mechanisms in the surrounding tissue^16,43-45^. Bergsmedh et al.^46^ demonstrated that tumor DNA may be horizontally transferred by the uptake of apoptotic bodies. Moreover, Burghoff et al.^44^ revealed that apoptotic bodies generated from transplanted HUVECs directly transmitted nucleic acids coding for EGFP to rat cardiomyocytes.

We isolated apoptotic bodies from different cell types (HUVECs, EPCs and fibroblasts) and transplanted them into the infarction borders, as described in our previous studies^8,10,13^. As expected, we did not observe significant changes in inflammatory cells. However, we also did not improvement in the hearts’ functional parameters. This finding was surprising, because immunohistochemistry revealed significant changes in neoangiogenesis among the groups. The number of vessels detected was comparable to that obtained by cell-transplantation in previous studies^8,10,13^, so the formation of new vessels alone does not suffice to improve the heart’s functional parameters in our model of chronical myocardial infarction. In the present context, the effects we have observed in the wake of EPC transplantation can only be explained by the inflammatory mechanisms.

However, the apoptotic bodies seem to contain minimal or undetectable protein levels of the main cytokines and chemokines. Their paracrine activity must be caused by something else. Therefore, it is very likely that their effect on surrounding cells is mediated by other mechanisms. As demonstrated by Burghoff et al.^44^ direct DNA transfer can be one of the mechanisms. However, since DNA sequences is random and its structure can not be defined inside of apoptotic bodies, the efficiency of transfer, correct integration, and consecutive transcription is low, difficult to control, and can not explain the reproducibility of the animal studies. We were able to show that apoptotic bodies contain miRNAs capable of paracrine actions. Extracellular miRNAs associated with lipid-based carriers and lipid-free proteins can be transferred from cell to cell and thereby affect gene expression^47^. We found a significant amount of miRNA in apoptotic bodies from each studied group. We also found significant differences between the apoptotic bodies isolated from each group. For example, HUVEC-AB and EPC-AB are similar in profile, but both are completely different from Fibro-AB. Although we found that several miRNAs known to induce or inhibit angiogenesis are expressed in all three cell lines, the pro-angiogenic miRNAs predominate in EPC-AB and HUVEC-AB, whereas in Fibro-AB the anti-angiogenic miRNAs predominate. This can in part explain the significant differences present in the histological results. Most of the miRNAs we detected, however, have not been characterized and their effect on angiogenesis or heart function is unknown.

Moreover, the mechanisms of cell transplantation in acute phase (directly) after myocardial infarction^48^ seem to be different compared to the mechanisms of cell transplantation in later phases (as in our model). Therefore, knowing precise factors involved in improvement of the heart function after cell therapy will help to identify the proper handling and use in each specific case of the cell-based therapy.

**Table 1:** Angiogenesis related miRNAs

miRNA	Angiogenesis	Target	Comments	
miR-132	increases	RasGAP	-activates the endothelium to facilitate pathological angiogenesis	^49^
miR-663	increases	VEGF	-critically for stress and oxidized lipids induced endothelial induction of transcription factor ATF4 and its downstream gene VEGF	^50^
miR-1908	increases		-endogenous promoters of metastatic invasion, angiogenesis, and colonization in melanoma	^51^
miR-23a	increases	Sprouty 2	-miR-23/27/24 cluster is involved in angiogenesis and endothelial apoptosis in cardiac ischemia and retinal vascular development	^52,53^
miR-222	increases	p27/Kip1	-induced tumor angiogenesis -induce proliferation and cell cycle progression	^54^
miR-199	increases		-endogenous promoter of metastatic invasion, angiogenesis, and colonization in melanoma	^51^
miR-24	decreases		-considerably upregulated after cardiac ischemia -induces endothelial cell apoptosis, abolishes endothelial capillary network formation -inhibition limited myocardial infarct size of mice via prevention of endothelial apoptosis and enhancement of vascularity	^52,55^
miR-29b	decreases/inhibits angiogenesis		-suppresses tumor angiogenesis, invasion, and metastasis by regulating matrix metalloproteinase 2 expression	^56,57^
miR-16	decreases tumor induced angiogenesis	VEGF, Bcl2	-induced tumor angiogenesis	^54^
miR-320	decreases/impaires angiogenesis	Flk-1, IGF-1, IGF-1R	-impaired angiogenesis in diabetic patients	^58^
miR-15b	decreases tumor induced angiogenesis	VEGF, Bcl2	-downregulated by hypoxia -overexpression induces apoptosis in leukemic cell line model -inhibition reduces number of cells G0/G1 promoting cell cycle progression	^54,59^
miR-221	decreases	c-kit, eNOS	-decreases EC-mediated angiogenesis -overexpression reduces tube formation, migration and wound healing in response to SCF	^53,54^

## Conclusion

**Figure 5:**
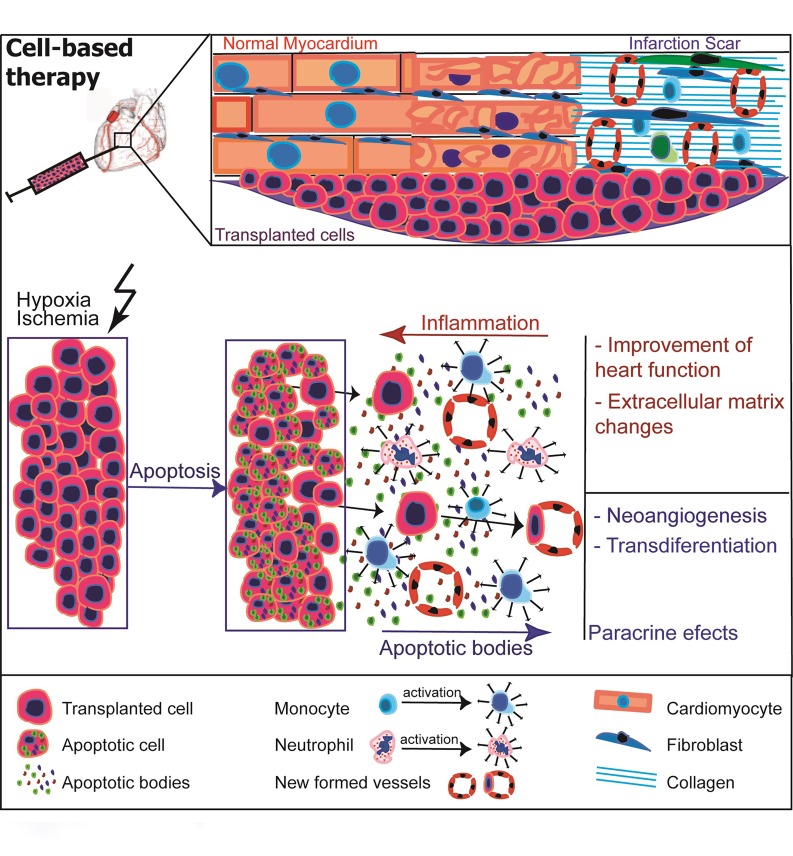
Cellular events after cell based therapy After transplantation, most of the cells undergo apoptosis. Apoptotic bodies are released and exert paracrine effects on the surrounding cells. At the same time, they activate resident macrophages, which can contribute to the remodelling of the extracellular matrix. On the other hand, the transplanted cells induce an inflammatory reaction, which supplementary recruit neutrophils and monocytes, crucial for the remodelling of the scar and improvement of the heart function. Only some of the cells integrate into the host tissue, without significantly affecting the heart function. Neoangiogenesis may be present, but is not a condition for improvement of heart function.

Summarizing our results (**Figure 5**), while improvement in myocardial function after cell-based therapy seems to be a result from a combination of mechanisms, the present study clearly identifies inflammation as critical for scar remodelling and improvement in heart function after later cell-based therapy. Although the paracrine effects of transplanted cells on their surrounding environment may not depend on inflammation and can induce changes in the extracellular matrix, and although their integration into host tissue can support resident cardiomyocytes, neither effect gives satisfactory results on heart function improvement. Neoangiogenesis can be substantially increased this way, but neoangiogenesis alone seems not to be sufficient to improve heart function. It is therefore imperative that researchers developing therapeutic strategies to improve remodelling and preserve heart function take account of the inflammatory effects.

- **CURRENT KNOWLEDGE: **Mechanisms of cell-based therapy are still not known; therefore, the therapy is inadequately translated in humans
- **AIM:** Elucidate cell-mediated regenerative mechanisms
- **DISCOVERIES:** Inflammatory reaction is critical for scar remodeling and improvement of the heart function after late cell-based therapy; Neoangiogenesis alone is not sufficient to improve heart function
